# Surgical treatment of an endobronchial lipoma obstructing the right upper bronchus: Imaging features with pathological correlation

**DOI:** 10.12669/pjms.296.3708

**Published:** 2013

**Authors:** Haiyong Wang, Zhenzong Du, Angui Li, Jianfei Song

**Affiliations:** 1 Haiyong Wang, MD, Department of Cardiothoracic Surgery, Affiliated Hospital of Guilin Medical College, Guilin 541001, China.; 2 Zhenzong Du, MD, Department of Cardiothoracic Surgery, Affiliated Hospital of Guilin Medical College, Guilin 541001, China.; 3 Angui LI, MD, Department of Cardiothoracic Surgery, Affiliated Hospital of Guilin Medical College, Guilin 541001, China.; 4 Jianfei Song, MD, Department of Cardiothoracic Surgery, Affiliated Hospital of Guilin Medical College, Guilin 541001, China.

**Keywords:** Endobronchial tumor, Lipoma, Bronchial obstruction, Surgical Resection, Lung Tumour

## Abstract

Endobronchial lipomas are rare benign unusual tumors of the respiratory tract. We describe a 65-year-old Chinese man with a history of cough due to an endobronchial tumor. The endobronchial biopsy was not excisional and was unable to evaluate the whole tumor. Then the mass was successfully resected via a right lateral thoracotomy. The histopathological diagnosis confirmed a benign lipoma arising from the membranous trachea. His CT features and fiberoptic bronchoscopic findings are shown along with the pathological results. In describing the management of this case, we stress that the clinical treatment of such tumors should be individualized according to the characteristics of each patient and mass.

## INTRODUCTION

Endobronchial lipomas are a relatively rare benign tumor accounting for only 0.1% of all lung neoplasms.^1^ Clinical symptoms of an endobronchial lipoma depend on the location of the mass, the severity of endoluminal obstruction, and the functional and anatomical effects on the lung distal to the obstruction. An endobronchial lipoma can lead to many symptoms including shortness of breath, cough, pneumonia and hemoptysis. Diagnosis is often misleading due to these non-specific symptoms and the appearance of the mass on imaging studies. We report a case of a 65-year-old man with an endobronchial lipoma causing obstruction of the right upper bronchus. 

## CASE REPORT

**Fig.1:**
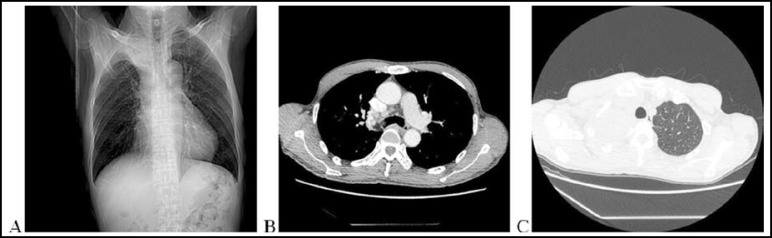
**(A) **Anterior-posterior chest X-ray showing atelectasis of the right upper lung. (**B**) Chest-computed tomography (CT) showing an extra-bronchial extension of tumor with low attenuation suggesting a fat containing lesion. **(C) **CT of the chest showing complete atelectasis of the right upper lobe secondary to bronchial obstruction (arrow).

A 65-year-old Chinese man presented with a sporadic, non-productive cough of two weeks’ duration. There was no history of fever, dyspnea, chest pain, hemoptysis or wheezing. On physical examination, he was found to have decreased breath sounds on the right chest. The patient had no outstanding medical history. Pulse oximetric saturation was 98% on room air. A chest radiograph showed atelectasis of the right upper lung (Fig.1A). A contrast enhanced chest CT showed a low attenuation mass in the right upper bronchus suggestive of a fat containing lesion (Fig.1B), with associated atelectasis in the right upper lobe (Fig.1C). There was no evidence of any enlarged mediastinal lymph nodes or pleural effusion. 

Exploration with a fiberoptic bronchoscope revealed a well-defined polypoid smooth-surfaced mass obstructing the right upper bronchus (Fig.3). The bronchoscope could not be advanced beyond the mass and into the right upper bronchus. Consequently, the right upper lobe bronchus was not visualized. A biopsy using a fiberoptic bronchoscope caused mass bleeding. Further Bronchoscopic resection was abandoned. Histopathological examination of the biopsies showed only acute inflammatory changes and did not allow evaluation of the whole tumor, surgical resection was arranged.

**Fig.2:**
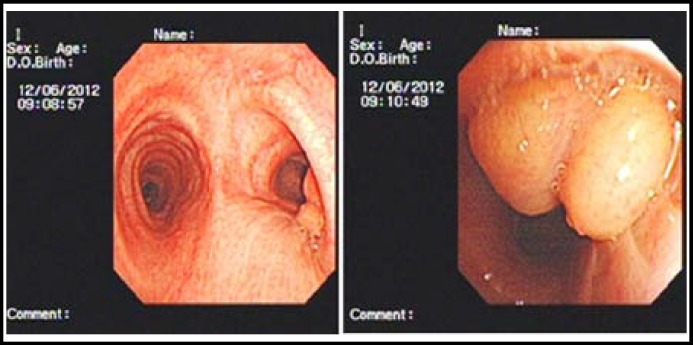
Endoscopic images showing a well defined, smooth tumor obstructing the right upper bronchus

**Fig.3:**
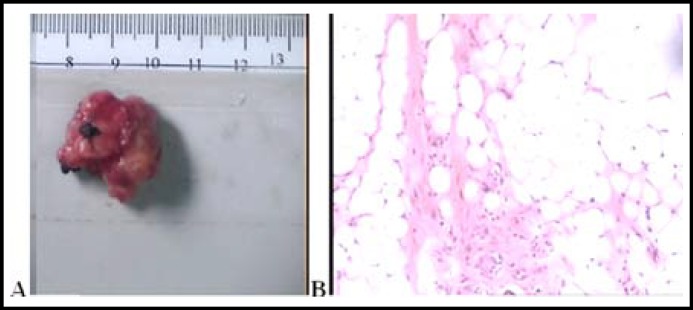
The mass was reddish and well circumscribed **(Fig.3A),** Microscopically, the tumor consists of lining respiratory epithelium and lobules of mature adipose tissue **(Fig 3B).**

During the ensuing right thoractomy, a slightly bulging part of the tumor was observed just under the right tracheobronchial angle as showed in Fig.2C. This was confirmed to be identical to the site of the intratracheal tumor located by fiberoptic bronchoscope. A wedge resection of the trachea, including the two cartilaginous rings containing the tumor, was performed. The defect was closed using interrupted absorbable 4-0 polyglyconate sutures, and the anastomotic site was thereafter buttressed with a pericardial fat pad flap. The right upper lobe was also resected. On pathological examination, the tumor was reddish and well circumscribed, measuring 2.2x1.8x1.1cm with a dumb-bell-shaped appearance (Fig.3A). On histological examination, the lesion consisted of mature adipose tissue (Fig.3B).

The patient was weaned from mechanical support immediately after the operation. On 11 month follow-up, the patient remained asymptomatic, with good clinical and radiological evaluations.

## DISCUSSION

Benign endobronchial tumors are uncommon, comprising fewer than 4% of all lung neoplasms.^2^ They are relatively rare benign neoplasms accounting for only 0.1% of lung tumors. Endobronchial lipomas occur more frequently in men with the median age of presentation being 60 years old. In most cases,^3^ the tumor has a predilection for the airway usually in a lobar or subsegmental location. These tumors are also more frequent on the right side—as in our present case - in which the tumor was in the right upper bronchus.

Clinical symptoms often occur late depending on the degree of airway obstruction, and cannot readily be distinguished from other causes of endoluminal obstruction. Common symptoms include cough, wheezing, and intermittent shortness of breath leading to a misdiagnosis of asthma or chronic obstructive pulmonary disease.

Theoretically, the sensitivity of a chest X-ray is low in diagnosing endobronchial tumors. Findings are often nonspecific and related to both postobstructive changes such as pleural effusions and atelectasis in symptomatic patients as well as bronchiectasis in asymptomatic patients. CT or MRI is an ideal tool to confirm the diagnosis. CT typically shows a homogeneous mass with fatty density and no tumor contrast enhancement.^4^ Similar results can be obtained by MRI. The findings on imaging often correlate with the tumor size and histopathology of the mass.

Bronchoscopy is indispensable to differentiate the fat content of the mass from malignancy or inflammation. Often, as in our case, one is unable to obtain correct information from which to make an accurate diagnosis through fiberoptic bronchoscope. Furthermore, bronchososopy resection may cause bleeding which, as in our case, obviates its utility.

Since most endobronchial lipomas are found after the onset of symptoms, postobstructive pneumonia can occur and result in chronic inflammation and destruction of the distal lung. The mainstay of treatment for endobronchial lipoma is, therefore, surgical or endoscopic resection. Early resection of benign endobronchial tumors may prevent distal lung damage. The method of resection (surgical or bronchoscopic) depends on the tumor size and the degree of lung damage. As a benign neoplasm, endoscopic treatment is now widely recommended as first line therapy. Bronchoscopic resection can achieve complete resolution of symptoms with low interventional risk compared to surgery.^5^

Optimal bronchoscopic resection indications for endobronchial lipoma include endoluminal tumors with limited extension into the endobrochial tree and a central locationn.^6^ But, if the tumor appears large, extraluminal extension, dumbbell-shaped on CT, or tumor dignity is uncertain, endoscopic procedures are not appropriate. Furthermore, if distal, destructive pulmonary changes due to the tumor are severe, the remaining peripheral lung will not recover after endoscopic procedures. In such cases, a surgical approach (lobectomy, wedge resection, or pneumonectomy) may be the therapy of choice. The prognosis is usually excellent in completely resected lesions.

Our case report embodies an important lesson in the management of patients with endobronchial lipoma patient. Despite the initial histology from bronchoscopy revealing only acute inflammatory changes, the final histology retrieved from the complete surgical resection showed a lipoma, which confirmed the CT findings.

## CONCLUSION

Endobronchial lipomas should initially be treated conservatively, but if the tumor has extraluminal extension or the distal destructive changes in the lung are severe, or if the endobronchial biopsy is not excisional and is unable to evaluate the whole tumor, surgical resection is necessary. The management of endobronchial lipoma should be individualized according to the characteristics of each patient, tumor anatomic factors and condition of the affected lung.

## Notes

***Consent: ***Written informed consent was obtained from the patient for publication of this paper and accompanying images. A copy of the written consent is available for review by the Editor in- Chief of this journal.
